# Ethnic inequalities among NHS staff in England: workplace experiences during the COVID-19 pandemic

**DOI:** 10.1136/oemed-2023-108976

**Published:** 2024-02-20

**Authors:** Rebecca Rhead, Lisa Harber-Aschan, Juliana Onwumere, Catherine Polling, Sarah Dorrington, Annahita Ehsan, Sharon A M Stevelink, Kamlesh Khunti, Ghazala Mir, Richard Morriss, Simon Wessely, Charlotte Woodhead, Stephani Hatch

**Affiliations:** 1 Psychological Medicine, King's College London - Institute of Psychiatry, Psychology & Neuroscience, London, UK; 2 Centre for Society and Mental Health, King's College London - Strand Campus, London, UK; 3 Demography Unit, Stockholm University, Stockholm, Sweden; 4 South London and Maudsley NHS Foundation Trust, London, UK; 5 King's Centre for Military Health Research, King's College London, London, UK; 6 Diabetes Research Centre, Leicester General Hospital, Leicester, UK; 7 Real World Evidence Unit, University of Leicester, Leicester, UK; 8 Leeds Institute of Health Sciences, University of Leeds, Leeds, UK; 9 Institute of Mental Health, University of Nottingham, Nottingham, UK; 10 NIHR ARC East Midlands, University of Nottingham, Nottingham, UK

**Keywords:** Ethnic Groups, Occupational Health, COVID-19, Health services research, Mental Health

## Abstract

**Objectives:**

This study aims to determine how workplace experiences of National Health Service (NHS) staff varied by ethnicity during the COVID-19 pandemic and how these experiences are associated with mental and physical health at the time of the study.

**Methods:**

An online Inequalities Survey was conducted by the Tackling Inequalities and Discrimination Experiences in Health Services study in collaboration with NHS CHECK. This Inequalities Survey collected measures relating to workplace experiences (such as personal protective equipment (PPE), risk assessments, redeployments and discrimination) as well as mental health (Patient Health Questionnaire (PHQ-9), Generalised Anxiety Disorder 7 (GAD-7)), and physical health (PHQ-15) from NHS staff working in the 18 trusts participating with the NHS CHECK study between February and October 2021 (N=4622).

**Results:**

Regression analysis of this cross-sectional data revealed that staff from black and mixed/other ethnic groups had greater odds of experiencing workplace harassment (adjusted OR (AOR) 2.43 (95% CI 1.56 to 3.78) and 2.38 (95% CI 1.12 to 5.07), respectively) and discrimination (AOR 4.36 (95% CI 2.73 to 6.96) and 3.94 (95% CI 1.67 to 9.33), respectively) compared with white British staff. Staff from black ethnic groups also had greater odds than white British staff of reporting PPE unavailability (AOR 2.16 (95% CI 1.16 to 4.00)). Such workplace experiences were associated with negative physical and mental health outcomes, though this association varied by ethnicity. Conversely, understanding employment rights around redeployment, being informed about and having the ability to inform redeployment decisions were associated with lower odds of poor physical and mental health.

**Conclusions:**

Structural changes to the way staff from ethnically minoritised groups are supported, and how their complaints are addressed by leaders within the NHS are urgently required.

**WHAT IS ALREADY KNOWN ON THIS TOPIC**

- Ethnically minoritised staff in the National Health Service (NHS) in England face significant workplace inequalities, including under-representation in senior roles and harassment.

**WHAT THIS STUDY ADDS**

- During the COVID-19 pandemic, black and mixed/other ethnic groups of NHS staff had higher chances of facing workplace harassment, discrimination and personal protective equipment unavailability, leading to negative health outcomes, but awareness of employment rights around redeployment and involvement in redeployment decisions were linked to better health outcomes.

**HOW THIS STUDY MIGHT AFFECT RESEARCH, PRACTICE OR POLICY**

- Urgent structural changes are needed to support minority ethnic staff in the NHS, including incorporating diversity and inclusion into professional development, involving staff in decision-making, educating them on their rights and expanding the NHS Workforce Race Equality Standard to ensure effective assessment of race equality.

## Introduction

Staff from ethnically minoritised groups constitute approximately 22% of the National Health Service (NHS) workforce in England (50% in London), but are under-represented in senior roles, more likely to face disciplinary action and experience less control over their working conditions compared with white staff.^1 2^ Furthermore, the NHS Workforce Race Equality Standard (WRES)—a programme designed to monitor race equality in the NHS—has consistently found that staff from ethnically minoritised groups experience disproportionate levels of discrimination and harassment from patients and colleagues (particularly the latter).^3 4^ Such experiences negatively impact mental and physical health and are associated with long periods of sickness absence.^5^ Qualitative research has also found that staff from ethnically minoritised groups working in London NHS Trusts may cope with bullying and microaggressions by moving teams or leaving their jobs.^2^

Within the UK, healthcare staff from ethnically minoritised groups have been over-represented in deaths due to COVID-19.^6–9^ This has also been seen in other countries.^10 11^ Reasons for this are complex but are partially the result of long-standing structural racism which has concentrated staff from ethnically minoritised groups in lower grades with more front-line work and greater exposure to COVID-19.^3^ Recent commentaries suggest staff from ethnically minoritised groups were more likely to be redeployed into hospital/clinic areas with a high risk of COVID-19 during the pandemic because they were unable to challenge or inform these decisions.^12^ Though COVID-19 risk assessments were introduced in April 2020 to ensure safe working conditions for all staff,^13^ these may have enabled further workplace inequalities if not conducted fairly.

Inequalities in COVID-19 exposure have also been compounded by disproportionately inadequate access to personal protective equipment (PPE). A survey of 1119 ethnically diverse UK healthcare staff during the pandemic found that 96% of ethnically minoritised participants believed that inadequate PPE had directly contributed to the transmission of COVID-19 in healthcare staff (vs 75% of White participants).^14^ Ethnically minoritised respondents were more likely to report concerns about PPE and to feel unable to decline requests to work in the absence of adequate PPE. These findings were echoed in a UK-based survey of 4418 nursing staff which found that staff from ethnically minoritised groups were more likely than white British staff to report problems accessing PPE, feel pressured to provide care without it, and have unaddressed PPE concerns.^15^ Similarly, UK-REACH (United Kingdom Research study into Ethnicity And COVID-19 outcomes in Healthcare staff) analysed data gathered between December 2020 and February 2021 from over 10 000 healthcare staff, finding that Asian ethnic staff groups were less likely to report access to adequate PPE compared with White staff groups.^16^ Finally, a qualitative study of 53 NHS staff and leaders, service users and community partners from ethnically minoritised backgrounds interviewed during the pandemic found that staff feared speaking up about working conditions would affect future employment. This was particularly true for agency/temp staff and those whose immigration status increased their precarity.^17^ Findings from these studies reflect the higher rates of COVID-19 and greater social risk factors for minority ethnic groups more widely.

Evidence suggests that pressurised working environments (eg, high workload, short staffing) can exacerbate bullying and discrimination.^2^ This may disproportionately impact staff from ethnically minoritised groups due to their over-representation at lower levels of the workforce hierarchy, negative stereotyping and prevailing organisational norms.^2^ Therefore, the extraordinary pressures of the COVID-19 pandemic may have potentially increased rates of bullying, harassment and discrimination for ethnically minoritised NHS staff, further impacting their mental and physical health.

NHS CHECK is one of the largest UK cohort studies conducted during the pandemic, established in April 2020 to longitudinally investigate the psychosocial impacts of the COVID-19 pandemic on NHS staff. The ongoing online survey assesses the mental health and well-being of NHS staff, students and volunteers within 18 NHS Trusts. Initial findings from NHS CHECK indicated that women, younger staff and nurses in London Trusts reported poorer mental health outcomes than other staff between April and June 2020.^18^ However, these analyses did not examine inequities by ethnicity.

Given the ongoing and evolving pressures beyond the COVID-19 pandemic, it is vital to recognise any persistent inequalities that may have led to negative health and job-related consequences for ethnically minoritised NHS staff. Thus, the Tackling Inequalities and Discrimination Experiences in Health Services (TIDES) study partnered with NHS CHECK to develop a survey to capture inequalities during the COVID-19 pandemic (the TIDES Inequalities Survey). Using data from this Inequalities Survey (cross-sectional), this paper aims to:

1. Estimate the prevalence of negative workplace experiences (eg, PPE unavailability, bullying) during the pandemic by ethnic groups.
2. Examine to what extent such experiences were associated with physical and mental health outcomes.

## Methods

The TIDES study investigates ethnic health inequalities and discrimination in UK health and social care providers (www.tidesstudy.com). Together with NHS peer researchers (healthcare staff trained in research methods) and national advisory and stakeholder opinion groups, TIDES codesigned an Inequalities Survey to be incorporated into the 10-month follow-up of NHS CHECK study. This survey was designed to assess the impact of COVID-19 on ethnic inequalities experienced by NHS staff.

**Figure 1:**
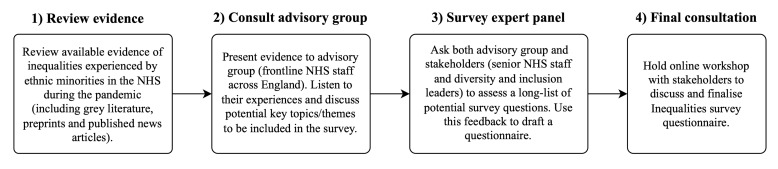
Consensus building process of working with stakeholders and advisory groups to produce the Inequalities Survey questionnaire. NHS, National Health Service.

The Inequalities Survey was compiled through a modified Delphi consensus process^19^ involving discussions and prioritisation surveys with front-line NHS staff, senior NHS managers/leaders and NHS equality, diversity and inclusion leaders (most of whom were from ethnically minoritised groups) about their experiences during the pandemic. The consensus-building approach was iterative, including several stakeholder discussions, piloting of proposed survey questions, and refining accordingly (figure 1). During these discussions, staff described experiences of PPE, poorly performed COVID-19 workplace risk assessments, sudden redeployments that could not be challenged or discussed, and experiences of discrimination and harassment in the workplace. All were described as disproportionately affecting ethnically minoritised staff. Questions on these topics and socioeconomic, occupational and COVID-19 related questions were included in the survey.

### Data

NHS CHECK invited all NHS staff (including medical, nursing, midwifery, allied health professionals, support, administrative, management, volunteers and students fast-tracked into clinical roles) working in 18 NHS Trusts across England (see online supplemental material A for the full list of Trusts) to participate in their study.^18^

10.1136/oemed-2023-108976.supp1 Supplementary data

The baseline NHS CHECK survey comprised two versions: a mandatory and expedited version, as well as an optional, more detailed version. However, both versions were relatively brief in nature. By contrast, subsequent follow-up surveys were characterised by greater length and complexity, including the incorporation of supplementary measures that were not present in the initial survey. In most participating Trusts, there was strong support for the baseline survey to be completed from senior NHS management as well as email and text reminders to staff. COVID-19 restrictions prevented researchers from gaining face-to-face access to staff and many front-line staff could not access email or personal phones in the workplace.

There were no monetary or other incentives to take part, but participants were entered into a prize draw. The NHS CHECK baseline sample consisted of 23 446 participants across 18 Trusts between April and December 2020 (total Trust population=139 037; response rate 5.9%).^20^ This was followed by a 6-month follow-up which was open between October 2020 and July 2021 (N=10 671; response rate 45.5%). All baseline participants were invited to participate in the Inequalities Survey (10-month follow-up, N=4622; response rate 19.7%) via email between February and October 2021 (3.3% of the total Trust population—see online supplemental figure). Participation in the Inequalities Survey involved completing an online questionnaire.

### Health outcomes

Measures to assess probable depression, anxiety and somatic symptoms were included in the survey. The Patient Health Questionnaire (PHQ-9) assessed depression, using a score of ≥10 to indicate probable depressive disorder (88% sensitivity and specificity for major depression).^21^ The Generalised Anxiety Disorder 7 (GAD-7) scale assessed anxiety, using a score of ≥10 to indicate probable anxiety disorder (89% sensitivity, 82% specificity).^22^ Both scales have very good to excellent levels of internal consistency and test–retest reliability.^23^ The PHQ-15 somatic symptom subscale assessed physical health,^24^ where 15 symptoms (eg, pain, nausea, fatigue) are rated as 0 (‘not bothered at all’), 1 (‘bothered a little’) or 2 (‘bothered a lot’), producing a score of 0–30. A score of ≥10 indicates moderate to severe somatic symptoms.

### Negative workplace experiences

Participants were asked if they had ever been unable to access PPE when at work during the pandemic and whether they had received a COVID-19 risk assessment. Participants were also asked if they had been redeployed during the pandemic and whether they had a good understanding of their employment rights relating to redeployment. If they had been redeployed, they were also asked if they were forewarned, or able to discuss or challenge the redeployment.

Discrimination was assessed by asking ‘In the last 12 months have you personally experienced discrimination at work from a manager/team leader or other colleagues’. The item assessing bullying, harassment and abuse (BHA) asked ‘In the last 12 months how many times have you personally *experienced* BHA from managers?’ and ‘In the last 12 months how many times have you personally experienced BHA from other colleagues?’. These items were combined and dichotomised to produce a single measure of whether the participant had experienced discrimination or BHA from any coworker. These measures of discrimination and BHA were taken from the NHS’s Staff Survey.^25^

### Analysis

Descriptive statistics described the Inequalities Survey sample by:

- Age (≤30, 31–40, 41–50, ≥51)
- Sex (male, female).
- Migrant status (yes, no).
- Region (London, South, North).
- Job role (doctor, nurse, other clinical, non-clinical).
- Employment contract (permanent contract, bank/agency shifts, both).
- Ethnicity (defined by UK Census categories,^26^ collected at baseline). Due to small sample sizes for specific ethnic groups (n<10), ethnicity was aggregated into five categories: white British, white other, black, Asian and mixed/other (see online supplemental material B for details of the ethnic groups included in these categories).

The prevalence of workplace experiences was calculated overall and for each ethnic group. Logistic regression was used to examine associations between (1) ethnicity and specific workplace experiences and (2) workplace experiences and mental health outcomes (probable anxiety, probable depression, moderate/severe somatic symptoms). These regression analyses estimated unadjusted ORs, and ORs adjusted for age, sex, region and month of survey completion, as well as confounders contract and job role (decided a priori and informed by relevant literature^2 5^). Additional subgroup analyses assessed the impact of BHA and discrimination experiences on probable depression for specific ethnic groups; sample size restrictions did not allow for subgroup analyses for other exposures and outcomes. Response weights were generated for the baseline NHS CHECK survey using iterative proportional fitting (a raking algorithm) based on age, gender, ethnicity and role. To use these weights, Inequalities Survey participants were treated as a subpopulation of the full (baseline) sample using the Survey packages’ subset command.^27^ This allowed our analysis to use the original design information from the baseline data but restrict survey design to participants of the Inequalities Survey. The alternative of dropping those who did not participate in the Inequalities Survey would produce correct estimates but incorrect SEs.^28^ Finally, postestimation commands from the Survey package were used to account for Trust size and response rate. Reported prevalence estimates, ORs and 95% CI were weighted, and frequencies were unweighted. All analysis was conducted in R V.4.2.0.^29^ using the Survey package.^27^

## Results

The demographic composition of the Inequalities Survey sample (n=4622) is similar to that of the baseline NHS CHECK survey. As shown in online supplemental material C, gender composition is the same across both samples and Inequalities Survey participants are slightly older. The composition of ethnic groups is broadly similar across both samples though the Inequalities Survey has a slightly higher percentage of staff who belong to the white other ethnic group (8.5% vs 6.3%) and slightly lower percentages of black (2.9% vs 4.3%) and Asian groups (4.8% vs 6.6%).

As shown in table 1, most of the sample were female (75%), born in the UK (84%), worked in clinical roles (68%) and had a permanent employment contract (90%). Almost half of the staff from black ethnic groups worked in non-clinical roles (compared with 33% in the white British group) and over two-thirds were London-based (compared with 13% in white British group). In contrast, staff from Asian ethnic groups were predominantly employed in clinical roles and had the highest proportion of doctors (20%), almost half were based in Northern England. Staff from the mixed/other group (mostly represented by mixed white and Asian groups) had the highest proportion of nurses (33%) among all other ethnic groups. Staff from white other groups had a similar composition of job roles as the white British group, but a higher proportion (82%) worked in the South/London region.

**Table 1:** Sociodemographic, work and health characteristics of Inequalities Survey participants by ethnicity

	Overall N=4622	White British n=3741	White other n=392	Black n=136	Asian n=220	Mixed/other n=133
Age (years) category						
≤30	645 (15.0%)	503 (14.4%)	51 (11.6%)	17 (12.4%)	44 (22.1%)	30 (26.1%)
31–40	923 (22.8%)	692 (21.0%)	112 (32.5%)	28 (21.1%)	69 (33.9%)	22 (14.2%)
41–50	1290 (25.6%)	1045 (25.5%)	102 (22.3%)	43 (28.7%)	60 (25.6%)	40 (36.0%)
51+	1764 (36.5%)	1501 (39.2%)	127 (33.6%)	48 (37.8%)	47 (18.4%)	41 (23.8%)
Gender						
Female	3725 (75.3%)	3050 (76.1%)	307 (69.8%)	107 (76.0%)	162 (73.6%)	99 (68.6%)
Male	825 (23.1%)	640 (22.3%)	77 (27.2%)	25 (21.4%)	53 (25.4%)	30 (29.4%)
Other	72 (1.7%)	51 (1.6%)	<11	<11	<11	<11
Migration						
UK born	3964 (83.8%)	3615 (96.9%)	91 (25.8%)	73 (51.2%)	97 (45.0%)	88 (54.5%)
Born outside UK	632 (16.2%)	105 (3.1%)	299 (74.2%)	61 (48.8%)	122 (55.0%)	45 (45.5%)
Job role						
Doctor	300 (9.3%)	200 (8.1%)	38 (11.9%)	<11	48 (20.0%)	<11
Nurse	1073 (27.7%)	868 (28.1%)	84 (27.5%)	38 (26.4%)	50 (24.5%)	33 (33.0%)
Other clinical	1263 (30.4%)	1018 (31.0%)	121 (30.4%)	31 (20.0%)	49 (30.1%)	44 (30.9%)
Non-clinical	1941 (32.5%)	1615 (32.8%)	146 (30.1%)	57 (45.3%)	73 (25.3%)	50 (32.4%)
Contract						
Permanent only	3522 (74.4%)	2926 (76.6%)	265 (68.0%)	88 (68.3%)	143 (66.4%)	100 (65.5%)
Permanent with some bank shifts	559 (15.7%)	417 (14.3%)	58 (18.0%)	27 (18.2%)	40 (21.6%)	17 (26.1%)
Bank shifts only	148 (3.0%)	109 (2.8%)	19 (4.5%)	<11	<11	<11
Other	352 (6.9%)	257 (6.3%)	45 (9.5%)	<11	30 (10.0%)	<11
Region						
London	1065 (20.5%)	638 (13.4%)	196 (42.3%)	89 (71.3%)	92 (29.4%)	50 (41.3%)
South	1951 (41.5%)	1697 (45.9%)	134 (39.6%)	16 (11.6%)	54 (24.4%)	50 (29.1%)
North	1606 (38.0%)	1406 (40.7%)	62 (18.1%)	31 (17.2%)	74 (46.2%)	33 (29.7%)
Unavailable PPE (if applicable)						
No	2756 (81.8%)	2260 (82.1%)	220 (78.6%)	69 (70.4%)	126 (90.0%)	81 (76.3%)
Yes	570 (18.2%)	450 (17.9%)	55 (21.4%)	27 (29.6%)	23 (10.0%)	15 (23.7%)
Risk assessment received						
No	931 (23.3%)	777 (24.3%)	94 (24.4%)	13 (11.2%)	24 (17.1%)	23 (33.5%)
Yes	2898 (76.7%)	2340 (75.7%)	231 (75.6%)	96 (88.8%)	143 (82.9%)	88 (66.5%)
Redeployed to another role						
No	2703 (65.4%)	2228 (65.5%)	213 (65.5%)	75 (65.9%)	111 (67.4%)	76 (54.1%)
Yes	1123 (34.6%)	886 (34.5%)	112 (34.5%)	34 (34.1%)	56 (32.6%)	35 (45.9%)
Experienced BHA from staff						
No	2738 (66.0%)	2252 (67.4%)	229 (64.3%)	65 (46.7%)	124 (70.8%)	68 (47.4%)
Yes	1253 (34.0%)	981 (32.6%)	111 (35.7%)	53 (53.3%)	59 (29.2%)	49 (52.6%)
Experienced discrimination from staff						
No	3254 (80.0%)	2711 (83.3%)	260 (74.6%)	68 (52.4%)	136 (77.1%)	79 (55.6%)
Yes	737 (20.0%)	522 (16.7%)	80 (25.4%)	50 (47.6%)	47 (22.9%)	38 (44.4%)
Probable depression (PHQ-9 score≥10)						
No	3015 (77.0%)	2469 (77.8%)	245 (73.1%)	85 (75.4%)	128 (78.0%)	88 (63.6%)
Yes	871 (23.0%)	687 (22.2%)	83 (26.9%)	28 (24.6%)	46 (22.0%)	27 (36.4%)
Probable anxiety (GAD-7 score≥8)						
No	3244 (82.4%)	2660 (83.3%)	264 (80.0%)	96 (86.8%)	129 (76.1%)	95 (72.0%)
Yes	642 (17.6%)	496 (16.7%)	64 (20.0%)	17 (13.2%)	45 (23.9%)	20 (28.0%)
Moderate/severe somatic symptoms (PHQ-15 score ≥10)						
No	2947 (76.6%)	2415 (77.0%)	235 (69.6%)	88 (76.9%)	131 (81.5%)	78 (66.7%)
Yes	922 (23.4%)	729 (23.0%)	92 (30.4%)	25 (23.1%)	40 (18.5%)	36 (33.3%)

Total cell counts may vary due to missing data.

Bank/agency shifts are temporary shifts at trust hospitals to cover staff absences. See online supplemental material A for full list of trusts.

BHA, bullying, harassment and abuse; GAD-7, Generalised Anxiety Disorder 7; PHQ, Patient Health Questionnaire; PPE, Personal Protective Equipmen.

Across the sample, 23% indicated probable depression, 18% indicated probable anxiety and 23% reported medium/severe somatic symptoms. Staff from the mixed/other ethnic group had a higher prevalence of probable depression, anxiety and somatic symptoms than all other ethnic groups (36%, 28% and 33%, respectively). One-third and one-fifth of all survey respondents reported experiences of BHA and discrimination, respectively (see table 1).

### Prevalence of workplace experiences by ethnicity

In table 2, findings indicate that staff from the black ethnic group had greater odds of receiving a risk assessment (adjusted OR 4.68, 95% CI 2.41 to 9.15) compared with staff from the white British group (see table 2). However, they also had greater odds of reporting PPE unavailability (adjusted OR 2.16, 95% CI 1.16 to 4.00). In contrast, staff from the Asian ethnic group had lower odds of reporting PPE unavailability (adjusted OR 0.38, 95% CI 0.20 to 0.72) compared with staff from the white British group. Staff from both black and mixed/other groups had greater odds of experiencing BHA (black: adjusted OR 2.43, 95% CI 1.56 to 3.78), mixed/other: adjusted OR 2.38, 95% CI 1.12 to 5.07) as well as discrimination from other staff members (black: adjusted OR 4.36, 95% CI 2.73 to 6.96, mixed/other: adjusted OR 3.94, 95% CI 1.67 to 9.33) compared with the white British group. Staff of white other ethnicity also had greater odds of experiencing discrimination (adjusted OR 1.61, 95% CI 1.10 to 2.35) compared with the white British group.

**Table 2:** Regression analysis to show associations between ethnicity and reported workplace experiences

Ethnicity	Unavailable PPE		Received risk assessment		Experienced BHA from staff		Experienced discrimination from staff	
	Crude OR (95% CI)	Adjusted OR (95% CI)	Crude OR (95% CI)	Adjusted OR (95% CI)	Crude OR (95% CI)	Adjusted OR (95% CI)	Crude OR (95% CI)	Adjusted OR (95% CI)
White other	1.24 (0.83 to 1.87)	1.04 (0.69 to 1.56)	1.00 (0.74 to 1.34)	1.31 (0.96 to 1.78)	1.15 (0.84 to 1.56)	1.14 (0.83 to 1.56)	1.70 (1.19 to 2.42)	1.61 (1.10 to 2.35)
Black	1.92 (1.14 to 3.24)	2.16 (1.16 to 4.00)	2.54 (1.33 to 4.84)	4.68 (2.41 to 9.11)	2.36 (1.57 to 3.57)	2.43 (1.56 to 3.78)	4.53 (2.96 to 6.93)	4.36 (2.73 to 6.96)
Asian	0.51 (0.27 to 0.96)	0.38 (0.20 to 0.72)	1.56 (0.73 to 3.32)	2.21 (1.01 to 4.84)	0.86 (0.54 to 1.36)	0.84 (0.52 to 1.33)	1.48 (0.91 to 2.42)	1.53 (0.93 to 2.52)
Mixed/other	1.42 (0.32 to 6.31)	1.22 (0.41 to 3.70)	0.64 (0.22 to 1.82)	0.86 (0.36 to 2.10)	2.29 (1.06 to 4.99)	2.38 (1.12 to 5.07)	3.99 (1.70 to 9.37)	3.94 (1.67 to 9.33)

Adjusted models adjust for age, sex, region, contract, job role and month of survey completion.

White British is the reference category.

BHA, bullying, harassment and abuse; PPE, personal protective equipment.

### Redeployment decision

35% (n=1123) of participants reported being redeployed into a different role during the pandemic (table 1). Of those who were deployed, staff from the black ethnic group had lower odds of feeling able to input into their redeployment (adjusted OR 0.58, 95% CI 0.28 to 1.20), while staff from the mixed/other group were less likely to be forewarned about their redeployment (adjusted OR 0.23, 95% CI 0.09 to 0.58, see table 3). Staff from the Asian ethnic group had greater odds of feeling able to challenge their redeployment decision (adjusted OR 3.17, 95% CI 1.26 to 7.99). Crude estimates approximate adjusted estimates (see online supplemental table).

**Table 3:** Regression analysis to show associations between ethnicity and redeployment experiences in those who were redeployed (n=1123)

Ethnicity	Able to challenge redeployment		Warned about redeployment		Able to have a say (input) about redeployment	
	n (%)	Adjusted OR (95% CI)	n (%)	Adjusted OR (95% CI)	n (%)	Adjusted OR (95% CI)
White British	508 (50.6)	—	650 (70.3)	—	489 (51.1)	—
White other	66 (56.9)	1.07	76 (67.3)	0.74	59 (49.4)	0.7
		(0.65 to 1.76)		(0.42 to 1.30)		(0.44 to 1.12)
Black	15 (42.5)	0.58	20 (62.1)	0.68	12 (31.5)	0.33
		(0.28 to 1.20)		(0.31 to 1.53)		(0.15 to 0.72)
Asian	35 (72.4)	3.17	41 (75.3)	1.46	35 (68.5)	2.38
		(1.26 to 7.99)		(0.59 to 3.62)		(0.95 to 5.97)
Mixed/other	15 (23.2)	0.37	21 (32.2)	0.23	18 (30.6)	0.52
		(0.14 to 0.94)		(0.09 to 0.58)		(0.21 to 1.33)

Adjusted models adjust for age, sex, region, contract, job role and month of survey completion.

Of all participants (regardless of whether they were redeployed or not), staff from the black ethnic group (35%, adjusted OR 0.53, 95% CI 0.32 to 0.86) were the only group less likely to understand their redeployment rights than white British staff (46%—see Online supplemental material D).

### Health outcomes by experience

As shown in table 4, unavailable PPE was associated with an approximately twofold increase in probable depression, probable anxiety and moderate/severe somatic symptoms. BHA and discrimination were also associated with an approximately threefold increase in each of these health outcomes. Conversely, understanding redeployment rights was associated with lower odds of probable depression and moderate/severe somatic symptoms.

**Table 4:** Regression analysis to show associations between workplace experiences and mental and physical health outcomes

Workplace experience		Probable depression (PHQ-9 score≥10)		Probable anxiety (GAD-7 score≥8)		Moderate/severe somatic symptoms (PHQ-15 score≥10)	
		n (%)	Adjusted OR (95% CI)	n (%)	Adjusted OR (95% CI)	n (%)	Adjusted OR (95% CI)
Unavailable PPE	Yes	192 (35.9)	2.01	136 (27.5)	1.73	1380 (34.0)	1.9
			(1.52 to 2.66)		(1.26 to 2.36)		(1.43 to 2.54)
	No	568 (20.6)	—	428 (15.9)	—	3907 (21.0)	—
Risk assessment	Yes	627 (22.2)	0.86	450 (16.8)	0.82	674 (22.4)	0.8
			(0.67 to 1.10)		(0.62 to 1.08)		(0.62 to 1.04)
	No	236 (26.0)	—	184 (20.2)	—	242 (27.3)	—
Discrimination	Yes	304 (43.8)	3.65	226 (34.8)	3.67	309 (41.8)	2.99
			(2.83 to 4.70)		(2.79 to 4.83)		(2.33 to 3.85)
	No	567 (17.8)	—	416 (13.4)	—	613 (18.8)	—
BHA	Yes	436 (35.7)	3.02	341 (28.7)	3.31	461 (36.5)	3
			(2.42 to 3.77)		(2.58 to 4.25)		(2.40 to 3.75)
	No	435 (16.4)	—	301 (11.9)	—	461 (16.6)	—
Redeployed	Yes	255 (23.4)	0.97	190 (18.4)	1	260 (24.0)	0.98
			(0.76 to 1.24)		(0.76 to 1.32)		(0.76 to 1.26)
	No	607 (22.9)	—	444 (17.2)	—	655 (23.2)	—
Understand redeployment rights	Yes	320 (18.5)	0.66	232 (14.6)	0.77	350 (18.3)	0.62
			(0.53 to 0.83)		(0.60 to 1.00)		(0.50 to 0.77)
	No	541 (26.8)	—	402 (20.0)	—	561 (27.6)	—
Able to challenge redeployment (if redeployed)	Yes	121 (19.2)	0.7	83 (14.3)	0.68	114 (18.5)	0.61
			(0.48 to 1.04)		(0.42 to 1.12)		(0.41 to 0.91)
	No	134 (27.8)	—	107 (22.7)	—	146 (29.9)	—
Warned about redeployment (if redeployed)	Yes	156 (18.9)	0.53	119 (15.7)	0.72	158 (20.1)	0.58
			(0.35 to 0.79)		(0.44 to 1.17)		(0.38 to 0.88)
	No	99 (33.2)	—	71 (24.3)	—	102 (32.9)	—
Able to have a say (input) about redeployment (if redeployed)	Yes	122 (18.3)	0.64	87 (14.4)	0.7	117 (17.9)	0.56
			(0.43 to 0.95)		(0.43 to 1.16)		(0.38 to 0.83)
	No	133 (28.6)	—	103 (22.5)	—	143 (30.4)	—

Adjusted models adjust for age, sex, region, contract, job role and month of survey completion.

BHA, bullying, harassment and abuse; GAD-7, Generalised Anxiety Disorder 7; PHQ, Patient Health Questionnaire; PPE, personal protective equipment.

Among those who were redeployed during the pandemic, having input into redeployment decisions and being forewarned about redeployment were associated with lower odds of probable depression and moderate/severe somatic symptoms. Being able to challenge redeployment decisions was also associated with lower odds of experiencing moderate/severe somatic symptoms. Crude estimates approximate adjusted estimates (see online supplemental table).

### Subgroup analysis

Descriptive subgroup analysis of probable depression by ethnicity, stratified by BHA and discrimination, indicated that across all ethnic groups, probable depression was more prevalent among those who reported these negative experiences, compared with those who did not (see online supplemental material E). However, the impact of these experiences on depression prevalence varied by ethnicity. Among those who did not experience BHA or discrimination at work, staff from the white British, Asian and black ethnic groups had the highest prevalence of depressive disorder. In contrast, among those who reported experiencing these negative experiences, staff from the white other and mixed/other ethnic groups had the highest prevalence of depressive disorder. However, due to small sample sizes, these finding cannot be generalised to the wider population.

## Discussion

Building on our prepandemic investigation into discrimination and inequalities in healthcare, this study aimed to identify ethnic inequalities in workplace experiences among NHS staff in England working during the COVID-19 pandemic. This work represents a collaborative effort with NHS CHECK and the NHS staff and leaders who comprised our advisory and stakeholder groups to inform the contents of our Inequalities Survey. Overall, this study found that negative workplace experiences such as discrimination, bullying and unavailable PPE were more likely to occur for staff from ethnically minoritised groups (particularly staff from black and mixed/other ethnic groups). These workplace experiences were associated with negative physical and mental health outcomes. Conversely, understanding employment rights around redeployment, being warned about an upcoming redeployment, and being able to inform redeployment decisions were associated with lower odds of poor health outcomes.

The study found that the difference in the likelihood of experiencing probable depression among those who faced bullying, harassment and discrimination varied by ethnicity. Specifically, the highest prevalence of depression was observed among the white other and mixed/other groups who had experienced discrimination and BHA. Conversely, among those who did not experience BHA, staff from the black and mixed/other ethnic groups had the highest prevalence of depression. However, these findings are based on small sample sizes, and thus, cannot be generalised. This highlights a broader issue where surveys often lack sufficient representation of ethnically minoritised groups to conduct effective subgroup analyses. To address this issue, academic researchers should prioritise building trust with communities to encourage participation in future studies. This can be achieved by involving community members and leaders in the survey design process, addressing concerns and offering incentives, providing transparency about the survey’s purpose and goals, partnering with trusted organisations and ensuring ethical standards are upheld throughout the survey process. Such actions can help researchers to build relationships with communities, demonstrate a commitment to their concerns and interests, and contribute to a more inclusive and equitable research process.

Experiences of BHA and discrimination from staff were highly prevalent in our study and substantially higher among staff from all ethnically minoritised groups compared with estimates in the 2022 NHS Staff Survey.^1^ The over-representation of London Trusts in the Inequalities Survey data may have contributed to these higher prevalence estimates, as London Trusts have been known to perform poorly on these measures.^1^ The external nature of the Inequalities Survey might have encouraged greater disclosure of experiences of workplace discrimination and BHA. This was the case for the prepandemic TIDES survey which found higher rates of BHA and discrimination compared with the NHS Staff Survey.^5^

Our research findings are consistent with previous studies, in the USA and the UK,^30^ including the UK-REACH, which identified disparities in PPE availability across different ethnic groups during the pandemic.^16^ Our study further contributes to the literature by demonstrating that inadequate PPE availability is associated with negative health outcomes among healthcare workers. This underscores the critical importance of ensuring equitable access to PPE (as well as a safe working environment for healthcare workers) during public health crises, particularly for healthcare workers from ethnically minoritised groups who are already vulnerable to health and socioeconomic inequities. Our findings underscore the urgent need for evidence-based policies and interventions that prioritise equitable distribution of PPE to all healthcare workers, irrespective of their demographic characteristics, to promote health and safety during public health emergencies.

Our study also found alarmingly high exposure to negative workplace experiences related to harassment and discrimination among ethnically minoritised NHS staff during the pandemic. These findings are consistent with the most recent NHS staff survey^1^ and UK-REACH^31^ in addition to being supported by multiple qualitative studies that have explored similar workplace experiences among ethnically and racially minoritised groups.^32–34^ The short-term and long-term impacts of such experiences are likely to take a toll on the mental and physical health of employees,^35^ as well as their dependents and social networks, with implications for career progression, intention to remain at the NHS and salary.^2^

### Strengths and limitations

The Inequalities Survey represents one of the largest surveys examining the impact of the pandemic on inequalities among healthcare staff. Despite targeted efforts to increase engagement, the response rate from those who participated in the NHS CHECK baseline study was low and varied by ethnicity. Specifically, the response rate by ethnicity was 21% for white British staff, 27% for staff from white other groups, 15% for staff from black ethnic groups, 15% for staff from Asian ethnic groups and 17% for staff from mixed/other ethnic groups. As a result, the relatively small sample sizes of staff from ethnically minoritised groups hindered our ability to examine the experiences of specific ethnic groups, such as black Caribbean nurses. Additionally, conducting a thorough subgroup analysis to estimate the mental health impact of workplace experiences on specific ethnic groups was hampered by the same issue of limited sample sizes.

These sample size issues are partly due to recruitment being limited to participants from the NHS CHECK baseline survey, which had an overrepresentation of NHS staff from White ethnic groups (NHS CHECK=86%, NHS workforce=78%). Survey fatigue may also have contributed. Poor response rates from ethnically minoritised groups reflect a wider issue with UK public health surveys which typically include a disproportionately large proportion of participants from white ethnic groups.^36^ As highlighted in a recent Wellcome report, ethnically minoritised groups have demonstrated greater levels of mistrust in research and health institutions during the pandemic.^37^ The over-representation of ethnically minoritised staff at lower professional grades could also impact their ability to complete the survey if they have less control over their working patterns. Ideally, to overcome this in future studies, staff from lower grades should be given protected paid time off for research participation. This would increase participation rates and improve the representation of under-represented groups in research studies.

Furthermore, the pandemic presented a particularly challenging context to recruit healthcare staff to research, given the stress experienced especially by ethnically minoritised staff. Nevertheless, a key strength of this survey was its tailored design to capture the unique experiences of ethnically minoritised NHS staff during these exceptional circumstances, by engaging staff and stakeholders through a consensus-building approach to improve representation. In addition, the data were weighted based on age, gender, ethnicity and role, using marginal sociodemographic data provided by participating trusts to ensure the sample better reflected our study population.

### Public health implications

The findings of this study provide additional evidence of the well-established link between institutional and interpersonal racism, structural inequalities and adverse health outcomes. It is crucial to prioritise racial discrimination as a public health issue, not just an ethical imperative and ensure that decision-makers from ethically minoritised groups are involved in processes that affect their health and well-being. This requires the acknowledgement of the systemic nature of racism, as well as the implementation of robust systems to combat its key mechanisms, such as racial discrimination, among ethnically minoritised staff.

Managers must also be trained to identify and handle reports of racial discrimination, with a shift in focus from generic cultural awareness and equality and diversity training, which has been found ineffective in tackling discrimination.^38^ Alternative approaches such as interactive or experiential training^39^ and inclusive leadership training,^40^ have been found to be more effective in addressing discrimination in the workplace. These approaches should also be incorporated into other professional development activities, such as leadership development programmes, onboarding processes and performance review systems.

Finally, this study identified health benefits for staff who understand their employment rights and are afforded opportunities to actively participate in decisions impacting their work. Consequently, NHS staff should be educated on their employment rights to ensure that they are able to advocate for themselves while also provided with adequate opportunities to engage in discussions, provide feedback and question decisions concerning their working conditions. To effectively facilitate and monitor progress towards these goals in a transparent manner, the NHS WRES may need to broaden its scope to include parameters such as tracking mechanisms for diversity and inclusion, as well as staff education initiatives. This would ensure that the NHS is actively monitoring and taking measures to improve in identified areas, while also ensuring that staff are equipped with the necessary knowledge and resources to create a more inclusive work environment. It would also aid in holding NHS leadership to account for addressing issues connected to diversity and inclusivity within their respective organisations.

## Conclusion

Against a backdrop of significant and publicised examples of health inequalities, discrimination and economic instability, NHS staff have navigated challenging working environments throughout the COVID-19 pandemic. Our findings suggest that staff from ethnically minoritised groups have also been exposed to greater harassment, discrimination and PPE unavailability than White British staff within the NHS, adding further burden to excess infection, mortality and need for intensive care among ethnically minoritised groups. Indeed, given the high number of key worker status staff within the NHS and their responsibility for providing healthcare, findings strongly suggest that NHS staff should be afforded greater protection and support throughout the pandemic and beyond.

Addressing these problems requires structural transformation in terms of how staff from ethnically minoritised groups are supported and how their complaints are addressed, including urgent policy attention and mandatory representation in institutional decision-making. Additionally, educating staff on their employment rights is crucial to ensure that they are aware of their rights and are able to advocate for themselves. These approaches are urgently required to address racism and inequalities in the UK healthcare system, which have long been recognised as both ‘avoidable and unjust’.

## Data Availability

Data are available on reasonable request. To request access to the data, please contact tides@kcl.ac.uk.
