# Supplementary material for: Ethnic inequalities among NHS staff in England: workplace experiences during the COVID-19 pandemic
Source: Occup Environ Med. 2024 Feb 20;81(3):113–21. doi: 10.1136/oemed-2023-108976 (PMC10958323; doi:10.1136/oemed-2023-108976)
Supplement: Supplementary data [file oemed-2023-108976supp001.pdf]

Ethnic inequalities experienced by NHS staff in England during the COVID-19 pandemic: Supplementary Material

Supplementary Material A: List of participating NHS Trusts 2
Supplementary Material B: Census grouping of ethnicities 3
Supplementary Material C: Sample composition comparison 4
Supplementary Material D: Understanding Employee Rights 5
Supplementary Material E: Subgroup analyses 6
Supplementary Tables: Table 3 and Table 4 (with both crude and adjusted estimates) 7
Supplementary Figure: Survey Timeline 9

Rhead R, *et al. Occup Environ Med* 2024; 81:113–121. doi: 10.1136/oemed-2023-108976

Supplementary Material A: List of participating NHS Trusts

The following NHS Trust participated in the NHSCHECK study.

**Trust names**
Avon & Wiltshire Mental Health Partnership NHS Trust
Cambridge University Hospitals NHS Foundation Trust
Cambridgeshire and Peterborough NHS Trust
Cornwall Partnership Trust
Devon Partnership NHS Trust
East Suffolk and North Essex NHS Trust
Gloucestershire Hospitals NHS Trust
Guy's and St Thomas' NHS Trust
King's College Hospital and PRUH
Lancashire and South Cumbria NHS Trust
Norfolk and Norwich University Hospitals
Nottinghamshire Healthcare NHS Trust
Royal Papworth Hospital
Sheffield Health and Social Care NHS Foundation Trust
South London and Maudsley (SLAM)
Tees Esk and Wear Valleys NHS Foundation Trust
University Hospitals of Derby and Burton
University Hospitals of Leicester NHS Trust

Supplementary Material B: Census grouping of ethnicities

Asian or Asian British

- Indian
- Pakistani
- Bangladeshi
- Chinese
- Any other Asian background

Black, Black British, Caribbean, or African

- Caribbean
- African
- Any other Black, Black British, or Caribbean background

Mixed or multiple ethnic groups

- White and Black Caribbean
- White and Black African
- White and Asian
- Any other Mixed or multiple ethnic background

White

- English, Welsh, Scottish, Northern Irish or British
- Irish
- Gypsy or Irish Traveller
- Roma
- Any other White background

Other ethnic group

- Arab
- Any other ethnic group

Rhead R, *et al. Occup Environ Med* 2024; 81:113–121. doi: 10.1136/oemed-2023-108976

Supplementary Material C: Sample composition comparison

	**Baseline (N = 22,834)**	**Inequalities Survey (N = 4,622)**
**Ethnicity**		
White British	18,127 (79%)	3,741 (81%)
White Other	1,446 (6%)	392 (9%)
Black	991 (4%)	136 (3%)
Asian	1,503 (7%)	220 (5%)
Mixed/Other	767 (3%)	133 (3%)
**Gender**		
female	18,487 (81%)	3,725 (81%)
male	4,232 (19%)	825 (18%)
other	109 (1%)	72 (2%)
**Age group (years)**		
≤30	4,367 (20%)	645 (14%)
31-40	5,068 (23%)	923 (20%)
41-50	5,794 (26%)	1,290 (28%)
51+	6,850 (31%)	1,764 (38%)
Unweighted %		

Rhead R, *et al. Occup Environ Med* 2024; 81:113–121. doi: 10.1136/oemed-2023-108976

Supplementary Material D: Understanding Employee Rights

Regression to show associations between ethnicity and understanding employee rights

	**n(%)**	**Crude OR [95%CI]**	**Adjusted OR [95%CI]**
White British	1,504 (46.3%)	—	—
White Other	156 (44.8%)	0.94 [0.70, 1.26]	0.94 [0.69, 1.28]
Black	39 (34.8%)	0.62 [0.40, 0.96]	0.52 [0.32, 0.85]
Asian	68 (37.5%)	0.70 [0.44, 1.11]	0.89 [0.56, 1.40]
Mixed/Other	50 (27.2%)	0.43 [0.20, 0.92]	0.49 [0.23, 1.06]

Adjusted models adjust for age, sex, region, contract, job role and month of survey completion

Rhead R, *et al. Occup Environ Med* 2024; 81:113–121. doi: 10.1136/oemed-2023-108976

Supplementary Material E: Subgroup analyses

Subgroup analysis of probable depression by ethnicity, stratified by BHA and discrimination

	No BHA<br>n (%)	BHA<br>n (%)	No Discrimination<br>n (%)	Discrimination<br>n (%)
**Probable depressive disorder**				
White British	350 (15.6)	337 (34.4)	467 (17.4)	220 (42.9)
White Other	35 (18.0)	48 (39.2)	50 (19.3)	33 (44.5)
Black	14 (21.7)	15 (25.6)	13 (18.1)	16 (30.1)
Asian	22 (14.1)	25 (37.1)	23 (13.8)	24 (45.2)
Mixed/Other	12 (22.8)	15 (46.8)	12 (19.2)	15 (55.7)

BHA = Bullying, Harassment or Abuse
Probable depressive disorder = PHQ-9 score of ≥10

Rhead R, *et al. Occup Environ Med* 2024; 81:113–121. doi: 10.1136/oemed-2023-108976

Supplementary Tables: Table 3 and Table 4 (with both crude and adjusted estimates)

Supplementary Table 3: Regression analysis to show associations between ethnicity and redeployment experiences in those who were redeployed (n=1,123) [BOTH CRUDE AND ADJUSTED ESTIMATES]

	Able to challenge redeployment			Warned about redeployment			Able to have a say (input) about redeployment		
**Ethnicity**	**n (%)**	**Crude OR [95%CI]**	**Adjusted OR [95%CI]**	**n (%)**	**Crude OR [95%CI]**	**Adjusted OR [95%CI]**	**n (%)**	**Crude OR [95%CI]**	**Adjusted OR [95%CI]**
White British	508 (50.6)	—	—	650 (70.3)	—	—	489 (51.1)	—	—
White Other	66 (56.9)	1.29 [0.81, 2.06]	1.07 [0.65, 1.76]	76 (67.3)	0.87 [0.53, 1.43]	0.74 [0.42, 1.30]	59 (49.4)	0.93 [0.59, 1.49]	0.70 [0.44, 1.12]
Black	15 (42.5)	0.72 [0.34, 1.52]	0.58 [0.28, 1.20]	20 (62.1)	0.69 [0.32, 1.49]	0.68 [0.31, 1.53]	12 (31.5)	0.44 [0.20, 0.95]	0.33 [0.15, 0.72]
Asian	35 (72.4)	2.56 [1.29, 5.08]	3.17 [1.26, 7.99]	41 (75.3)	1.29 [0.60, 2.74]	1.46 [0.59, 3.62]	35 (68.5)	2.08 [1.03, 4.20]	2.38 [0.95, 5.97]
Mixed/Other	15 (23.2)	0.30 [0.09, 0.95]	0.37 [0.14, 0.94]	21 (32.2)	0.20 [0.06, 0.68]	0.23 [0.09, 0.58]	18 (30.6)	0.42 [0.13, 1.41]	0.52 [0.21, 1.33]

Adjusted models adjust for age, sex, region, contract, job role and month of survey completion

Supplementary Table 4: Regression analysis to show associations between workplace experiences and mental and physical health outcomes [BOTH CRUDE AND ADJUSTED ESTIMATES]

		Probable Depression (PHQ-9 score ≥10)			Probable Anxiety (GAD-7 score ≥8)			Moderate/Severe Somatic Symptoms (PHQ-15 score ≥10)		
**Workplace Experience**		**n (%)**	**Crude OR [95%CI]**	**Adjusted OR [95%CI]**	**n (%)**	**Crude OR [95%CI]**	**Adjusted OR [95%CI]**	**n (%)**	**Crude OR [95%CI]**	**Adjusted OR [95%CI]**
Unavailable PPE	Yes	192 (35.9)	2.15 [1.62, 2.87]	2.01 [1.52, 2.66]	136 (27.5)	2.01 [1.44, 2.80]	1.73 [1.26, 2.36]	1,380 (34.0)	1.90 [1.42, 2.54]	1.90 [1.43, 2.54]
	No	568 (20.6)	—	—	428 (15.9)	—	—	3,907 (21.0)	—	—
Risk assessment	Yes	627 (22.2)	0.81 [0.63, 1.04]	0.86 [0.67, 1.10]	450 (16.8)	0.80 [0.60, 1.06]	0.82 [0.62, 1.08]	674 (22.4)	0.77 [0.59, 0.99]	0.80 [0.62, 1.04]
	No	236 (26.0)	—	—	184 (20.2)	—	—	242 (27.3)	—	—
Discrimination	Yes	304 (43.8)	3.59 [2.79, 4.62]	3.65 [2.83, 4.70]	226 (34.8)	3.45 [2.61, 4.56]	3.67 [2.79, 4.83]	309 (41.8)	3.10 [2.42, 3.98]	2.99 [2.33, 3.85]
	No	567 (17.8)	—	—	416 (13.4)	—	—	613 (18.8)	—	—
BHA	Yes	436 (35.7)	2.84 [2.26, 3.55]	3.02 [2.42, 3.77]	341 (28.7)	2.97 [2.31, 3.81]	3.31 [2.58, 4.25]	461 (36.5)	2.88 [2.31, 3.59]	3.00 [2.40, 3.75]
	No	435 (16.4)	—	—	301 (11.9)	—	—	461 (16.6)	—	—
Redeployed	Yes	255 (23.4)	1.02 [0.80, 1.30]	0.97 [0.76, 1.24]	190 (18.4)	1.09 [0.82, 1.43]	1.00 [0.76, 1.32]	260 (24.0)	1.05 [0.82, 1.33]	0.98 [0.76, 1.26]
	No	607 (22.9)	—	—	444 (17.2)	—	—	655 (23.2)	—	—
Understand redeployment rights	Yes	320 (18.5)	0.62 [0.50, 0.77]	0.66 [0.53, 0.83]	232 (14.6)	0.68 [0.53, 0.88]	0.77 [0.60, 1.00]	350 (18.3)	0.59 [0.47, 0.73]	0.62 [0.50, 0.77]
	No	541 (26.8)	—	—	402 (20.0)	—	—	561 (27.6)	—	—
Able to challenge redeployment (if redeployed)	Yes	121 (19.2)	0.62 [0.42, 0.92]	0.70 [0.48, 1.04]	83 (14.3)	0.57 [0.35, 0.91]	0.68 [0.42, 1.12]	114 (18.5)	0.53 [0.35, 0.80]	0.61 [0.41, 0.91]
	No	134 (27.8)	—	—	107 (22.7)	—	—	146 (29.9)	—	—
Warned about redeployment (if redeployed)	Yes	156 (18.9)	0.47 [0.30, 0.73]	0.53 [0.35, 0.79]	119 (15.7)	0.58 [0.35, 0.97]	0.72 [0.44, 1.17]	158 (20.1)	0.51 [0.33, 0.80]	0.58 [0.38, 0.88]
	No	99 (33.2)	—	—	71 (24.3)	—	—	102 (32.9)	—	—
Able to have a say (input) about redeployment (if redeployed)	Yes	122 (18.3)	0.56 [0.38, 0.83]	0.64 [0.43, 0.95]	87 (14.4)	0.58 [0.36, 0.93]	0.70 [0.43, 1.16]	117 (17.9)	0.50 [0.33, 0.75]	0.56 [0.38, 0.83]
	No	133 (28.6)	—	—	103 (22.5)	—	—	143 (30.4)	—	—

Adjusted models adjust for age, sex, region, contract, job role and month of survey completion
PPE = Personal Protective Equipment
BHA = Bullying, Harassment or Abuse

Supplementary Figure: Survey Timeline

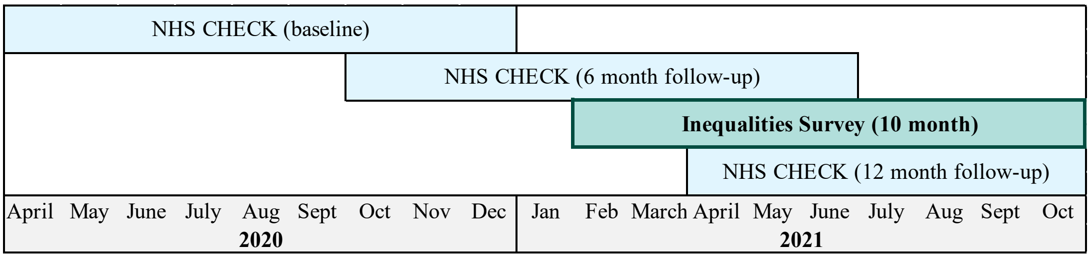

Supplementary Figure: Timelines for the TIDES Inequalities Survey alongside NHS CHECK surveys

Rhead R, *et al. Occup Environ Med* 2024; 81:113–121. doi: 10.1136/oemed-2023-108976
